# AAV2/8-hSMAD3 gene delivery attenuates aortic atherogenesis, enhances Th2 response without fibrosis, in LDLR-KO mice on high cholesterol diet

**DOI:** 10.1186/s12967-014-0252-8

**Published:** 2014-09-20

**Authors:** Hongqing Zhu, Maohua Cao, Jose A Figueroa, Everado Cobos, Barry F Uretsky, Maurizio Chiriva-Internati, Paul L Hermonat

**Affiliations:** Central Arkansas Veterans Healthcare System, 111J, 4300 West 7th Street, Little Rock, AR 72205 USA; Department of Internal Medicine, Division of Hematology & Oncology, Texas Tech University, Health Sciences Center, School of Medicine, Lubbock, TX 79415 USA; Kiromic LLC, Lubbock, Texas USA

**Keywords:** Atherosclerosis, Gene therapy, Human mothers against decapentaplegic homolog 3 (hSMAD3), Transforming growth factor beta 1 (TGF β 1), Adeno-associated virus 2/8 (AAV2/8), Fibrosis, CD68, EMR, Collagen, Connective tissue growth factor (CTGF)

## Abstract

**Background:**

Inflammation is a key etiologic component in atherogenesis and transforming growth factor beta 1 (TGFβ1) is a well known anti-inflammatory cytokine which potentially might be used to limit it. Yet TGFβ1 is pleiomorphic, causing fibrosis, cell taxis, and under certain circumstances, can even worsen inflammation. SMAD3 is an important member of TGFβ1′s signal transduction pathway, but is a fully intracellular protein.

**Objectives:**

With the hope of attenuating TGFβ1′s adverse systemic effects (eg. fibrosis) and accentuating its anti-inflammatory activity, we proposed the use of human (h)SMAD3 as an intracellular substitute for TGFβ1.

**Study design:**

To test this hypothesis adeno-associated virus type 2/8 (AAV)/hSMAD3 or AAV/Neo (control) was tail vein injected into the low density lipoprotein receptor knockout (LDLR-KO) mice, then placed on a high-cholesterol diet (HCD).

**Results:**

The hSMAD3 delivery was associated with significantly lower atherogenesis as measured by larger aortic cross sectional area, thinner aortic wall thickness, and lower aortic systolic blood velocity compared with Neo gene-treated controls. HSMAD3 delivery also resulted in fewer aortic macrophages by immunohistochemistry for CD68 and ITGAM, and quantitative reverse transcriptase polymerase chain reaction analysis of EMR and ITGAM. Overall, aortic cytokine expression showed an enhancement of Th2 response (higher IL-4 and IL-10); while Th1 response (IL-12) was lower with hSMAD3 delivery. While TGFβ1 is often associated with increased fibrosis, AAV/hSMAD3 delivery exhibited no increase of collagen 1A2 or significantly lower 2A1 expression in the aorta compared with Neo-delivery. Connective tissue growth factor (CTGF), a mediator of TGFβ1/SMAD3-induced fibrosis, was unchanged in hSMAD3-delivered aortas. In the liver, all three of these genes were down-regulated by hSMAD3 gene delivery.

**Conclusion:**

These data strongly suggest that AAV/hSMAD3 delivery gave anti-atherosclerosis therapeutic effect without the expected undesirable effect of TGFβ1-associated fibrosis.

## Background

TGFβ1 is an important anti-inflammatory cytokine and is protective against various cardiac pathologies. It has been identified to be protective against myocardial ischemia-reperfusion injury [1,2], apoptosis of cardiomyocytes [3], pro-inflammatory adhesion molecule expression on the vascular endothelial cells [4], and foam cell formation [5]. It also inhibits ox-LDL-induced expression of adhesion molecules in endothelial cells [6]. Furthermore, it is well established that TGFβ1 is a strong inhibitor of immune response [7,8]. Taken together, TGFβ1 would seem to be a logical candidate for an effective anti-atherogenesis gene. In a previous study TGFβ1^ACT^ gene delivery has shown efficacy against atherosclerosis in the low density lipoprotein receptor knockout (LDLR-KO) mice on high-cholesterol diet (HCD) [9]. While we did not observe significant adverse effects upon TGFβ1^ACT^–gene delivery into LDLR-KO/HCD animals, we did find certain higher cytokine levels. Moreover, higher TGFβ1 expression is well known to be linked to adverse reactions such as fibrosis, infection, cancer and increased infections, coupled with unwanted immune effects (reviewed in [10]).

Another approach to use TGFβ1 therapeutically might be to utilize the genes down-stream from TGFβ1 in its signal transduction pathway(s), through which TGFβ1 acts. Previously, for example, we have shown that STAT3 gene delivery, down-stream of interleukin 10 (IL-10), can substitute for IL-10 gene delivery in the inhibition of atherosclerosis in a mouse model [11]. These downstream genes would then code for intracellular proteins, usually transcription factors, and have a more limited effect than a systemically secreted protein. The mechanism would be that such genes might enhance the effects of low level endogenous TGFβ1, and take the place of direct TGFβ1^ACT^ gene delivery [9]. Thus, hopefully, by going through the most desirable TGFβ1^ACT^ signal transduction pathway, undesirable side effects (eg. fibrosis) would be avoided. The anti-inflammatory abilities of TGFβ1 work through a number of signal transduction pathways, including Ras-ERK, TAK1-JNK Rho-Rac-cdc42, mothers against decapentaplegic homologs (SMADs) 2, 3, 4, and others [9]. While SMAD2, SMAD3 or SMAD4 might all be proposed as substitute agents for TGFβ1, perhaps the most interesting and appropriate of these agents may be SMAD3 [12]. SMAD3 knockout (KO) mice have a phenotype that closely mimics that of the TGFβ1 KO mouse [13]. Moreover, SMAD3 KO mice display much higher and systemic inflammatory cell infiltration [12]. While many studies suggest TGFβ1 acts through SMAD3/SMAD4, other studies suggest SMAD3 can act without SMAD4, and can even compete with SMAD4 for binding both protein and DNA targets [14]. This is likely due to SMAD3′s ability to either homodimerize or heterodimerize with SMAD4.

Thus we hypothesized that SMAD3 might be a reasonable intracellular substitute for TGFβ1 with a more specific signal transduction effect. It is unclear at what level SMAD3 is basally expressed in endothelial and smooth muscle cells, but it is clear that SMAD3 can be induced in these cells [15,16]. Thus, by inducing higher constitutive SMAD3 levels in cardiovascular tissues, in particular, aortic smooth muscle cells (the main target of adeno-associated virus in arteries, AAV) [17], we should be able to enhance the effects of endogenous secreted TGFβ1. Adeno-associated virus (AAV), first investigated in 1984, is a useful tool for gene delivery to study gene function / therapeutic effect [18-21], and its expression is known to last at least 10 years in clinical trials [22]. The predicted amino acid (aa) sequence homology of mouse and human (h) SMAD3 is 99%, thus, hSMAD3 was the choice as a therapeutic gene. In this study we delivered the hSMAD3 gene using AAV type 8 (AAV8) capsid, which has been shown to be effective in gene delivery into cardiovascular tissues by ourselves and others [23-27]. Here we observed that AAV/hSMAD3 delivery resulted in efficacy in inhibiting atherosclerosis in low density lipoprotein receptor knockout (LDLR-KO) mice on high cholesterol diet (HCD), but without the concomitant fibrosis associated with TGFβ1.

## Methods

### AAV vector construction and virus generation

The human (h) SMAD3 and calcitonin gene-related protein (CGRP) cDNAs (obtained from Open Biosystems) were ligated downstream from the cytomegalovirus immediate early promoter within the gutted AAV vector dl3-97 to generate AAV/hSMAD3 and AAV/hCGRP, respectively. The AAV/Neo vector has been described previously [28]. AAV2/8 virus (AAV2 DNA in AAV8 virion) was produced using pDG8 helper (TransIT transfection of 6 μg each of pDG8 plus AAV vector plasmid into 10 cm plates of 293 cells), freeze-thawing the plates three times at 60 hours, the virus concentrated (pelleted) by ultracentifugation, and titered by dot blot hybridization analysis by standard methodologies [29].

#### Animal treatments

LDLR KO mice (B6;129S7-*Ldlr*^*tm1Her*^/J) were purchased from Jackson Laboratories (Bar Harbor, ME, USA). Three groups of male mice, composed of ten animals each at 8 weeks old, were injected with AAV/Neo (positive control group), AAV/hSMAD3 or AAV/hCGRP virus at a titer of 1 × 10^10^ eg/ml via tail vein with 200 μl virus per mouse, two booster injections were followed at an interval of 5–6 days. High cholesterol diet (HCD) of 4% cholesterol and 10% Coco butter diet (Harlan Teklad, Madison, Wis, USA) was provided from the first day of injection and maintained for the entire study period. Another group of mice fed with a normal diet was used as a negative control group. All experimental procedures conform to protocols approved by the Institutional Animal Care and Usage Committee of the Central Arkansas Veterans Health Care System at Little Rock.

#### Ultrasound imaging

The Vevo 770 High-Resolution Imaging system (Visualsonics, Toronto, Canada) with a RMV 707B transducer was used for all direct aortic examinations as described earlier [24,26,27,30]. Briefly, each mouse was anesthetized with inhalation of 1.5% isoflurane (Isothesia, Abbot Laboratories, Chicago, USA) with oxygen and placed supine on a thermostatically heated platform to maintain a constant body temperature. All legs were taped to ECG electrodes for cardiac function monitoring. Abdominal hair was removed using a chemical hair remover (Church & Dwight Co., Inc., NJ, USA), pre-warmed US gel (Medline Industries, Inc., Mundelein, USA) was spread over the skin as a coupling medium for the transducer. Image acquisition was started on B-mode; two levels of the vessel were visualized longitudinally: thoracic region and renal region. Thereafter, a short-axis view was taken to visualize the same arterial site in a cross-sectional view immediately. For each level, individual frames and cine loops (300 frames) were acquired and recorded at distances of 1 mm throughout the length of the aorta. Measurements and data analysis was performed off line using the customized version of Vevo 770 Analytical Software from both the longitudinal and transverse images.

#### Tissue sampling, processing, and immuno-histochemistry

At 20 weeks after first injection of virus and on HCD, mice were euthanized by CO2 exposure and exsanguinations to collect blood. For immunohistochemistry analysis was prepared as described earlier [6,29,30]. The aorta was flushed with saline solution and fixed in 10% neutral-buffered formalin (Sigma, St Louis, MO, USA). After 24 hrs, the fixed tissue was embedded by paraffin for sectioning. After immunohistochemistry staining with anti-CD68 or anti-ITGAM antibody with conjugated horse radish peroxidase, photomicrographs were taken. For real-time PCR analysis, the aorta sample were frozen in liquid nitrogen and stored in −80°C.

#### Measurement of plasma cholesterol

Total plasma cholesterol of AAV/Neo and AAV/SMAD3 mice were measured by VetScan VS2 (Abaxis, Union City, CA, USA) at the Veterans Animal Laboratory (VAMU).

### RNA isolation and real-time qRT-PCR

Total aortic RNAs were extracted using Trizol reagent (Invitrogen Carlsbad, CA) and were treated with DNase I (Invitrogen, Carlsbad, CA). Then cDNA was synthesized using oligo(dT)_18_ primers and RNase H-reverse transcriptase (Invitrogen, Carlsbad, Calif) according to the manufacturer’s instructions. The specific primers for qPCR analysis were synthesized by Integrated DNA Technologies, Inc. (Coralville, IA). Real-Time Quantitative PCR was performed using SYBR Green PCR Master Mix kit on the Applied Biosystems Fast 7900HT real-time PCR system (Applied Biosystems, Foster City, CA). The results were analyzed with SDS 2.3 software. Table 1 lists the primer set sequences used.

**Table 1:** **Primer sequences**

**GENE**	**polarity**	**5′ 3′**
βactin	sense	ATCTGGCACCACACCTTCTAC
	antisense	GAAGGTCTCAAACATGATCTGG
COL1A2	sense	CGTTCCCAAAGATGGTAGAT
	antisense	AGCCACCTCCGCTGACACCA
COL2A1	sense	GAACATCACCTACCTACCACTG
	antisense	ATCCTTCAGGGCAGTGTA
CTGF	sense	GGAGAAGCAGAGCCGCCT
	antisense	CTGGTGCAGCCAGAAAGC
EMR	sense	AAGTATTCCAACTGCTCT
	antisense	ATTGGCCCTCCTCCACTA
IL-4	sense	ATGTGCCAAACGTCCTCACA
	antisense	AGAACACTAGAGTTCTTCTT
IL-7	sense	AAATGACAGGAACTGATAGT
	antisense	GACATTGAATTCTTCACTGA
IL-10	sense	TACAGCCGGGAAGACAATAA
	antisense	AAGGAGTCGGTTAGCAGTAT
IL-12	sense	AGAATCACAACCATCAGCAG
	antisense	TTCACTCTGTAAGGGTCTGC
ITGAM	sense	TCCAGAGGCTGTGAATAT
	antisense	CTTCTGAAAGTCAATGTTGT
SMAD3	sense	GGTCCCTGGATGGCCGGT
	antisense	GGATTCACGCAGACCTCG

Shown are the indicated primer sets used to analyze gene expression by QRT-PCR.

#### Western blot analysis

Tissue samples were collected after the mice were sacrificed. Proteins were extracted from liver with the T-PER tissue protein extraction reagent (Thermo Scientific). Protein quantification was measured by the protein assay dye reagent kit (Bio-RAD). Samples were loaded at equal amount and were electrophoresed on 10% SDS-PAGE gels and transferred to immune-blot PVDF membrane (Bio-Rad). After blocking with 5% nonfat milk in 1 × TBST buffer (10 mM Tris-Cl [pH 7.5], 150 mM NaCl, 0.1% Tween 20), membranes were incubated at room temperature for 1 hour with a monoclonal antibody specific to the SMAD3 (1:1000 dilution, Sigma-Aldrich) or a monoclonal antibody specific to β-actin (1:1000 dilution, Sigma-Aldrich). Membrane was then washed with 1 × TBST buffer (10 mM Tris-Cl (pH 7.5), 150 mM NaCl, 0.1% Tween 20) and incubated with 1:2000 dilution horseradish peroxidase-conjugated secondary antibody (Sigma-Aldrich) at room temperature for 1 hour. Proteins were detected using the ECL system (Fisher-Scientific) and exposure to X-Ray film (Phenix).

## Results

### HSMAD3 gene delivery and protein expression

**Figure 1:**
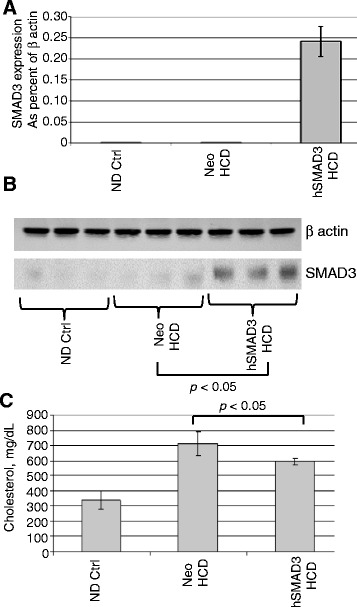
**Delivery of hSMAD3 and dietary effects. A**. Relative expression of the hSMAD3 gene to βactin by real-time quantitative PCR from aorta of 3 mice in each group. For qRT-PCR the quantity of RNA for each gene was normalized to βactin in the same sample. Data shown are mean +/− SE. **B**. shows a western blot analysis of protein from liver probed with anti-SMAD3 antibody. Note that both **A** and **B** show increased SMAD3 levels in the AAV/hSMAD3-treated animals. **C**. shows the levels of total cholesterol. Note that a HCD did result in increased cholesterol.

We address the hypothesis that hSMAD3 gene can serve as a substitute for TGFβ1. AAV/hSMAD3 (AAV serotype 8) was delivered by tail vein injection and the animals placed on HCD (4% cholesterol, 10% coco butter). The animals were then harvested/high resolution ultrasound analyzed at 16–20 weeks post-injection/post HCD initiation. shows that the delivery of hSMAD3 into the aorta, as measured by Q-RT-PCR, was successful, being expressed much higher in AAV/hSMAD3-treated animals. A western blot analysis, in Figure 1B, was done for SMAD3 protein in the liver of the various animal groups and it can be seen that AAV/hSMAD3 infected animals had higher protein levels (*p* <0.05). It should be noted that the primers used in Figure 1A were specific for the hSMAD3 gene present in the AAV vector, while the western blot utilized antibody which identified both endogenous mouse SMAD3 and AAV gene therapy-derived human SMAD3. Figure 1C shows that the blood cholesterol levels were high in both groups on HCD, but that the AAV/hSMAD3-treated animals were statistically lower. Additionally, animal weights were statistically similar in all groups (data not shown).

### Analysis of aortic structure

**Figure 2:**
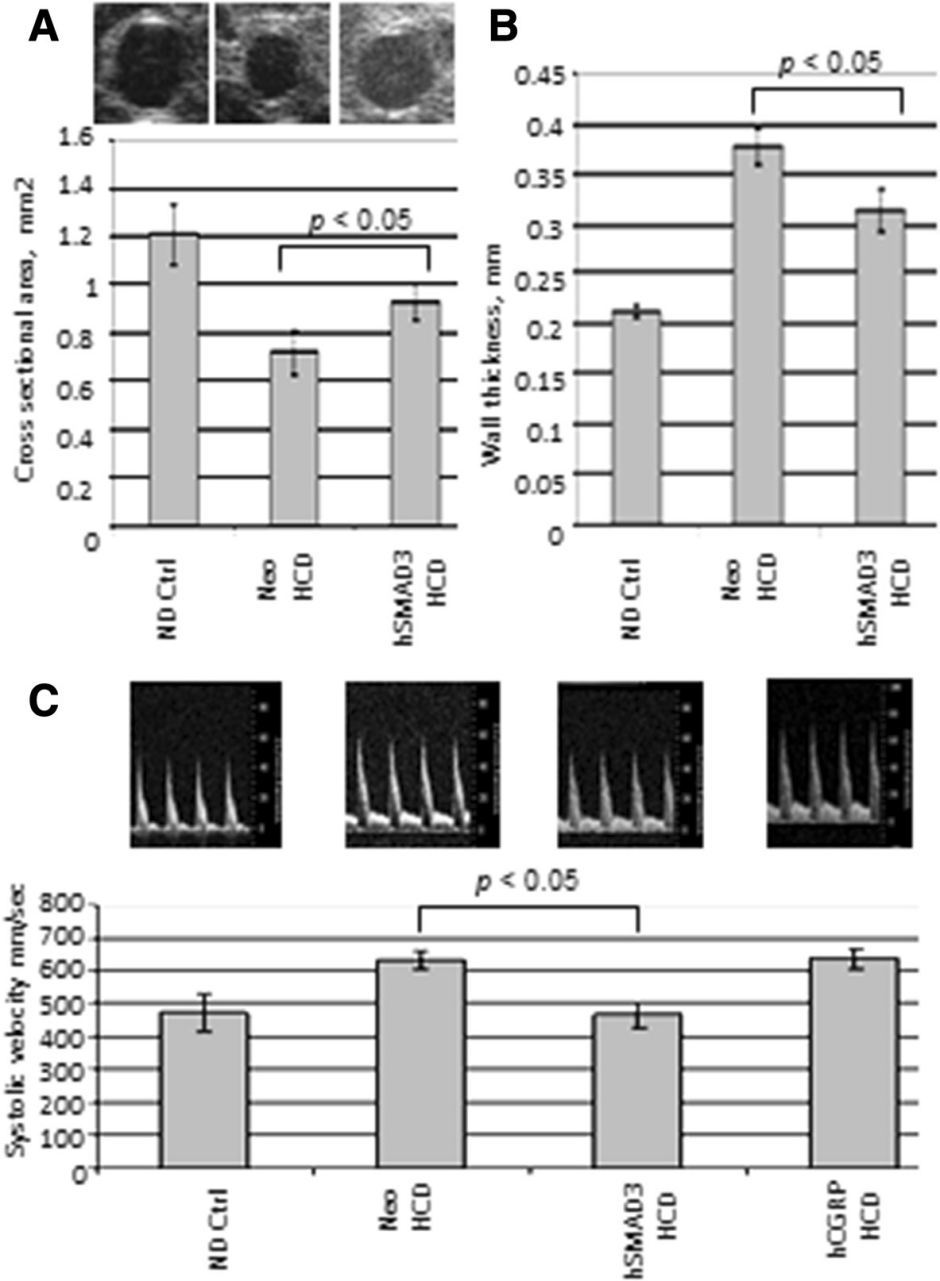
**Structural Analysis of the aorta.** High resolution ultrasound (HRUS) was used to measure various aortic parameters. **A**. shows quantification of the cross-sectional area for the thoracic region of the aorta in 3–5 animals from each animal group by HRUS with representative captured images from the analysis just above. Note that the AAV/hSMAD3-HCD animals had a larger cross sectional area than the AAV/Neo-HCD animals. **B**. shows quantification of the wall thickness of the aorta (thoracic region). Note that the AAV/hSMAD3-HCD animals have a thinner wall thickness than the AAV/Neo-HCD animals. **C**. shows quantification of blood flow velocities in the lumenal center of the abdominal region of the aorta in 3–5 animals from each group with representative captured images from the analysis just above. Note that the AAV/hSMAD3-HCD animals have a much lower blood velocity than the AAV/Neo-HCD animals (or hCGRP-treated).

High resolution ultrasound (HRUS) was then used to analyze the aortas of at least eight animals per group. Figure 2A shows that the aortic cross-sectional area was significantly larger (*p* < 0.05) in the hSMAD3/HCD-treated animals than the Neo/HCD-treated animals by HRUS. Moreover, HRUS, as shown in Figure 2B, indicated that aortic wall thickness was significantly lower (*p* < 0.05) in the hSMAD3/HCD-treated animals than the Neo/HCD-treated animals, consistent with lower atherosclerosis. Figure 2C shows that the systolic blood velocity in the thoracic region of the aorta was significantly lower (*p* < 0.05) in the hSMAD3/HCD-treated animals than the Neo/HCD-treated animals, consistent with less severe atherosclerosis. In sharp contrast to the effect of hSMAD3 delivery, another gene we thought might have some therapeutic effect, calcitonin gene-related peptide (CGRP), had no effect on systolic aortic blood velocity. Thus, CGRP will not be further studied. However, importantly, the results from the aortic cross-sectional area, wall thickness and systolic blood velocity were all consistent, all indicating there was significantly lower atherosclerosis associated with hSMAD3 gene delivery.

### Analysis of macrophage trafficking

**Figure 3:**
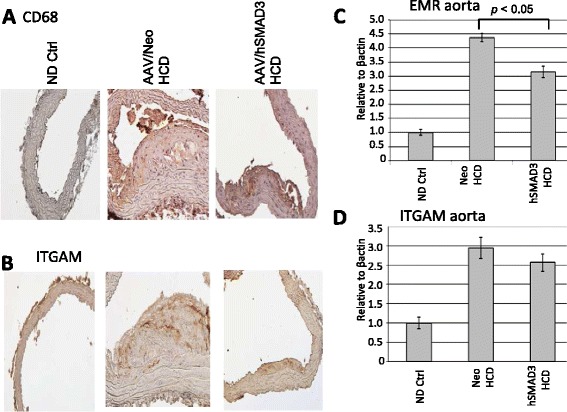
**Macrophage burden of aortic tissue. A**. CD68 expression. CD68 is a marker of macrophages and thus is a general marker of inflammation. Histologic sections of aorta from the indicated animal groups were analyzed for CD68 protein by immunohistochemistry using anti-CD68 antibody. Note that the AAV/hSMAD3-HCD-treated animals displayed a much lower brown CD68 signal than the AAV/Neo-HCD-treated animals strongly suggesting lower inflammation. **B**. shows a similar analysis with anti-ITGAM antibody, another marker of macrophages, with similar results to CD68. **C**. shows a QRT-PCR analysis of EMR expression, another macrophage marker. Note, again, macrophage levels were significantly lower (*p* <0.05) in hSMAD3-treated animals than Neo-treated. **D**. shows a QRT-PCR analysis of ITGAM expression. Note, again, macrophage levels trended lower in hSMAD3-treated animals than Neo-treated.

**Figure 4:**
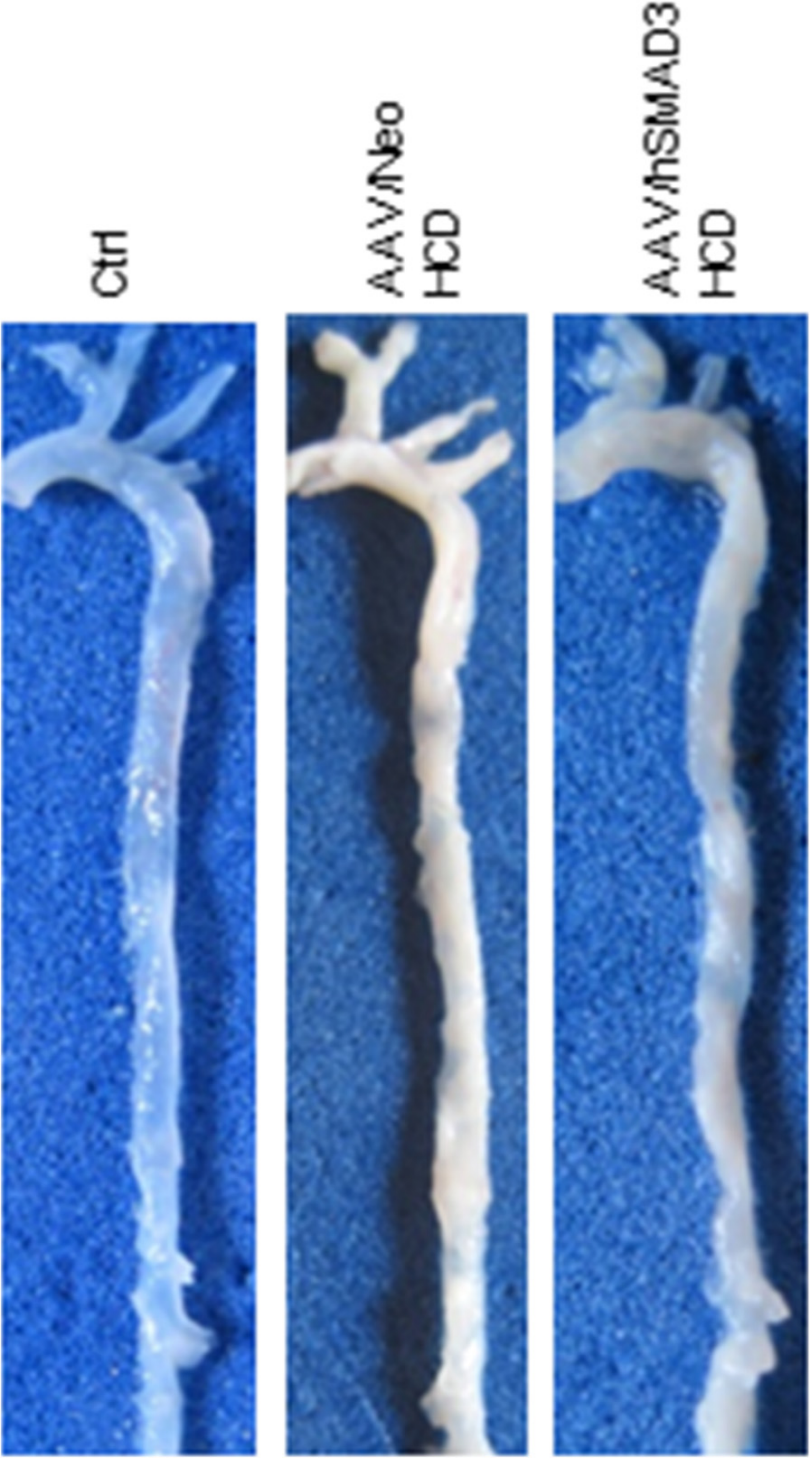
**Visual inspection of representative aortas.** Aortas from the indicated animals were buffered formalin fixed, cleaned and photographed. Note that the AAV/Neo-treated HCD aorta displays much higher amounts of lipid accumulation (white areas) than the AAV/hSMAD3-treated-HCD animals.

The level of macrophage trafficking into the aortic wall was analyzed by immune-histochemistry using anti-CD68- and anti-ITGAM antibody, as shown in Figure 3A and 3B, respectively. For both macrophage markers there was shown to a greater number of macrophages in the walls of the Neo/HCD-treated than the SMAD3/HCD-treated animals. Macrophage invasion of the aorta was also quantified by QRT-PCR for the expression of another macrophage marker, EMR. As shown in Figure 3C, the level of EMR in the SMAD3/HCD-treated animals was significantly lower (p <0.05) than in Neo/HCD-treated animals. QRT-PCR analysis of ITGAM, as another Mo/Mac marker, also trended lower (*p* = 0.67), consistent with the immune-histochemistry data. Thus, these data, taken together, indicate that macrophage levels are lower SMAD3/HCD-treated animals than in Neo/HCD-treated animals. Representative cleaned and unstained aortas are also shown in Figure 4. Areas of plaque, high cholesterol, show up as white areas, whereas normal aorta is translucent. These aortas show that the AAV/Neo-HCD-treated aorta had higher levels of lipid accumulation in contrast to either the ND ctrl or to the AAV/SMAD3-HCD-treated aorta (no quantification performed).

#### Immune status of the aortas

**Figure 5:**
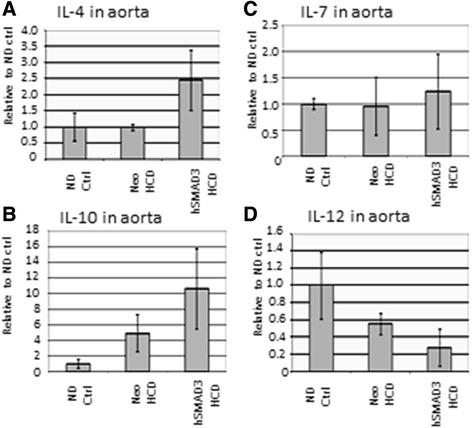
**Immune response status of aortas is Th2. A**. shows a QRT-PCR analysis of IL-4 expression, a Th2 response cytokine. Note that IL-4 levels were significantly higher (*p* <0.05) in hSMAD3-treated animals than Neo-treated. **B**. shows a QRT-PCR analysis of IL-10 expression, another Th2 response cytokine. Note, again, IL-10 levels trended higher in hSMAD3-treated animals than Neo-treated. **C**. shows a QRT-PCR analysis of IL-7 expression, a Th1 response cytokine. Note that IL-7 levels were higher (*p* <0.05) in hSMAD3-treated animals than Neo-treated, however, overall, the changes were very minor. **D**. shows a QRT-PCR analysis of IL-12 expression, another Th1 response cytokine. Note IL-12 levels trended lower in hSMAD3-treated animals than Neo-treated.

Related to macrophage and lipid accumulation, we observed the expression of various Th1 and Th2 cytokines in the aortas in order to determine the aortas’ predominant immune status. Figure 5A shows that Th2 cytokine IL-4 was significantly (*p* >0.05) higher in SMAD3/HCD-treated animals than in the Neo/HCD-treated animals. Similarly, IL-10 levels, another Th2 cytokine, trended higher in the SMAD3/HCD-treated animals (Figure 5B). In contrast, IL-7, a Th1 cytokine, was unchanged in all group (Figure 5C), while IL-12 (Figure 5D), yet another Th1 cytokine, trended lower SMAD3/HCD-treated animals over Neo/HCD-treated animals. Thus, overall, these data establish that a predominant Th2 response is present in the aortas as a result of the SMAD3 delivery, consistent with the known overall effect of TGFβ1.

### Analysis of collagen expression/fibrosis

**Figure 6:**
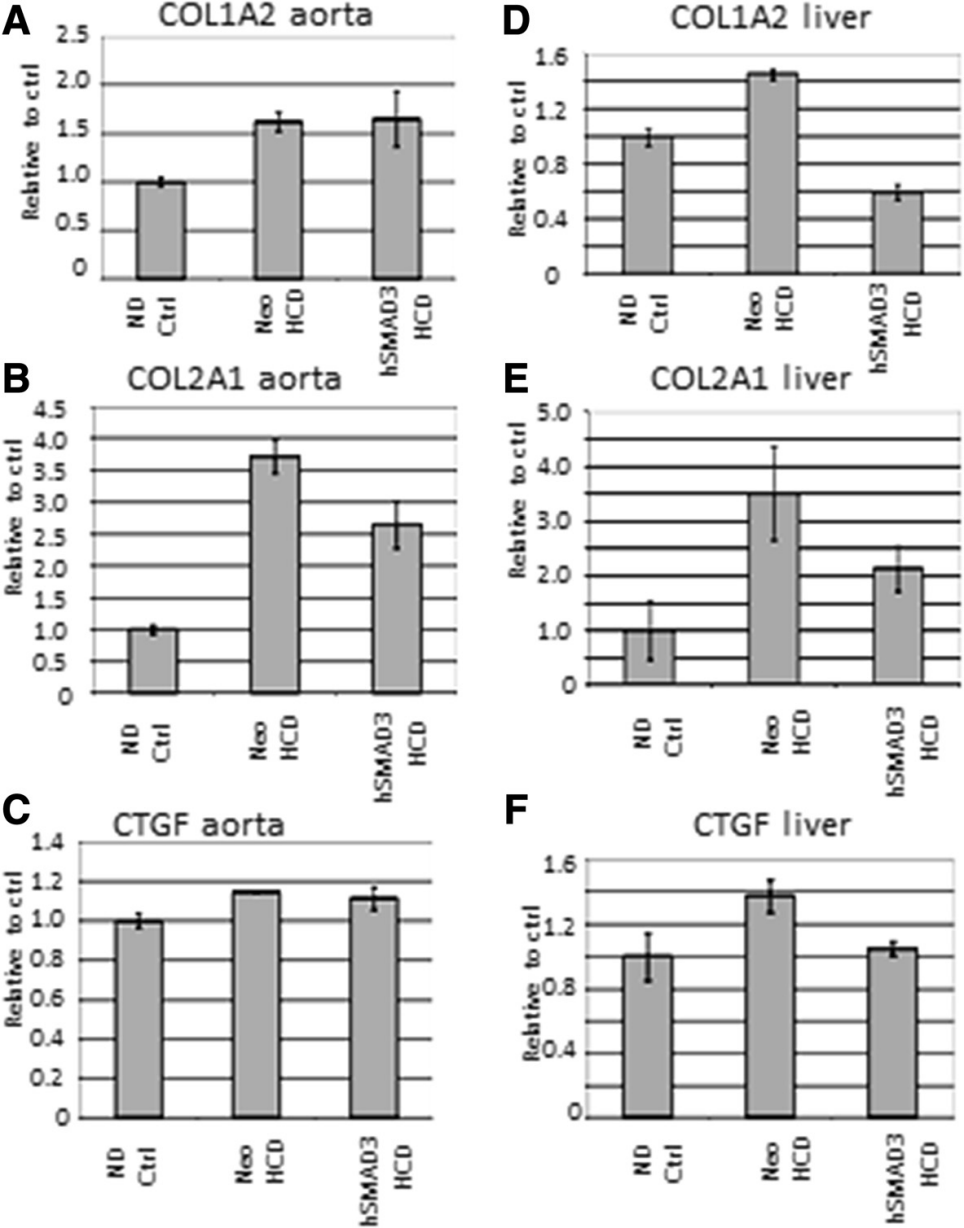
**Collagen (COL) and connective tissue growth factor (CTGF) expression in the aorta and liver. A**. shows a Q-RT-PCR analysis of COL1A2 expression in the aortas, a major marker of fibrosis. Note that COL1A2 levels were essentially the same in the aortas of both hSMAD3-HCD- and Neo-HCD-treated animals. **B**. shows a Q-RT-PCR analysis of COL2A1 expression in aortas, another marker of fibrosis. Note that COL2A1 levels were significantly lower in the aortas of both hSMAD3-HCD-treated than Neo-HCD-treated animals. **C**. shows a Q-RT-PCR analysis of CTGF expression in the aortas, again, another marker of fibrosis. No significant change is seen in any experimental group. **D**, **E**, and **F** show an analogous Q-RT-PCR analysis of COL1A2, COL2A1, and CTGF expression, respectively, but this time in the liver. Note that all three genes are significantly down-regulated by hSMAD3 delivery compared to Neo control, fully consistent with lower fibrosis.

Fibrosis is one of the most undesirable side effects of TGFβ1 expression. Thus, the possibility that SMAD3 might be useful as a TGFβ1-substitute would hinge on its ability to give therapeutic effect, without association with adverse side effects. With our atherosclerosis model we can only study the TGFβ1 side effects of increased cancer and infections with great difficulty. However, status of fibrosis would be easy to study. Therefore, in Figure 6A, the level of collagen 1A2 expression was analyzed by QRT-PCR and no significant difference in expression in the aortas was found between hSMAD3/HCD- and Neo/HCD-treated animals. However, collagen 2A1 expression (Figure 6B) was significantly lower in hSMAD3/HCD- than Neo/HCD-treated animals. The lack of increased fibrosis was further substantiated by observing connective tissue growth factor (CTGF) expression, a known inducer of fibrosis, was unchanged throughout all groups (Figure 6C). The effects of AAV/hSMAD3-delivery were further analyzed directly in the liver, a known target for fibrosis, as well as a known target for AAV8-based gene delivery. In an analogous analysis to the aorta, collagen 1A2, collagen 2A1 and CTGF were all significantly down in AAV/hSMAD3-HCD-treated animals in Figure 6D-F. In summary, Figure 6 demonstrates that AAV/hSMAD3-delivery is associated with lower fibrosis, not higher fibrosis, as is with TGFβ1.

## Discussion

The TGFβ1/SMAD signaling pathway has been shown to mediate immunosuppressive responses. This fact suggests that SMAD3, one member of these down-stream genes, might limit atherogenesis through its anti-inflammatory effects. However, TGFβ1/SMAD3 signaling is also pro-fibrotic [10]. Using AAV-based gene delivery, we studied which of these effects was dominant *in vivo* and if hSMAD3 might be an intra-cellular, non-systemic substitute for TGFβ1 in treating/inhibiting atherogenesis. As defined by larger aortic lumen, thinner aortic walls, by lower systolic blood velocity, and lower macrophage burden, hSMAD3-gene delivery clearly resulted in a significant anti-atherogenic effect. While AAV8-delivered hSMAD3 expression was only approximately 0.3% mRNA expression compared to endogenous βactin expression, assuming that the average aortic cell (smooth muscle) translates the βactin and the hSMAD3 mRNAs at roughly the same efficiency and are roughly equivalent to the average cultured fibroblast [31], then a 0.3% expression level appeared to result in, approximately 400 hSMAD3 molecules per minute per cell produced. However, this analysis doesn’t address that the transduction level of AAV8 after 20 weeks (less than 100%), nor the likelihood that smooth muscle cells express more βactin than fibroblasts. Thus the level of hSMAD3 being produced specifically in AAV-transduced cells is likely to be multiples higher than the 400 molecules per minute per cell.

The level of hSMAD3 expression clearly affected intra-aortic immune response. We observed that IL-4 was significantly up, as expected, suggesting that Th2 response is favored over Th1 response. IL-10, another Th2 cytokine, also trended higher, in agreement with IL-4. Correspondingly, IL-12, a Th1 cytokine trended lower in the hSMAD3-treated liver, consistent with higher Th2 cytokines. The expression of IL-7, was technically higher in hSMAD3-treated aortas, but actually levels were little changed. Thus, overall, the delivery of hSMAD3 resulted in a pro-Th2 intra-aortic response, as is known for TGFβ1 [32], and is believed beneficial for countering atherogenesis [33].

While SMAD3 is most often linked with TGFβ1 signaling, there are other ligands which also involve SMAD3 in their signal transduction pathways. One example is activin, another member of the TGFβ super family. Its effects are on the reproductive system, insulin and muscle metabolism, and enhancement of fibrosis [34]. However, these pathways are not as extensively studied as TGFβ1. Additionally, angiotensin II (Ang II), a well known blood pressure regulator of the renin-angiotensin system, also signals though SMAD3, but in a less direct manner. Ang II signaling through AT1R, down-regulates SMAD7, a negative regulator of SMAD3, and results in higher SMAD3 signaling. Yet, again, fibrosis is enhanced by Ang II [35]. Other ligands, such as inhibin may also signal through SMAD3. Thus, SMAD3′s role in signal transduction has been described in the title of one review article as having “smaddening complexity” [36].

The purpose of using hSMAD3 gene delivery was to provide the therapeutic effect of TGFβ1, but with lower adverse effects of fibrosis, cancer and infections. While we can only study cancer and infection levels with great difficulty in our model, we can more readily study fibrosis. A major finding of this study was that collagen and connective tissue growth factor (CTGF) expression was unaffected or lower in the aorta, and significantly lower in the liver, after AAV/hSMAD3 delivery (Figure 6). We were surprised to find collagen 1A2 expression was the same in Neo-HCD and hSMAD3-HCD-treated aortas. Supporting these data, collagen 2A1 was significantly higher in Neo-HCD than in hSMAD3-HCD-treated aortas. Consistent with these data, CTGF, known to induce SMAD3-associated fibrosis, was unchanged throughout all groups. Even more telling, collagen 1A2, collagen 2A1, and CTGF expression were significantly lower in hSMAD3-HCD-treated livers. There is evidence that SMAD3, as it lowers fibrosis, may also lower cancer. SMAD3 knockout mice develop various cancers and SMAD3 expression is known to be cell cycle regulated by ras [37]. Thus, a simple explanation may be that our gene delivery of constitutive SMAD3 expression gives a constant anti-proliferative effect, with fibrosis being down-regulated along with cell proliferation. Leivonen *et al*. [38] found that SMAD3 is specifically required (not SMAD2 or SMAD4) for the induction of matrix metalloproteinase-13 (MMP-13) by TGFβ1 [38]. Higher MMP-13 levels are also associated with lower fibrosis [39]. This latter signaling pathway and phenotype is also more consistent with what we observe.

It must also be mentioned that our results are in contrast with those of Kundi *et al*., [40], who found increased fibrosis after adenovirus-based gene delivery of SMAD3, following carotid injury in rats [40]. However, the adenovirus vector used in the Kundi study is well known to cause inflammation, NFkB induction and fibrosis during gene therapy experiments as well as clinically [41-44]. Thus, when issues of inflammation and fibrosis are possible the use of adenoviral vectors would not seem to be ideal. It should also be mentioned our results are consistent with those of Meng *et al*. 2012 [13], who found that SMAD3-dimers could translocate to the nucleus without SMAD4, and that SMAD3 were defective in activating the COL1A2 promoter (and perhaps others). As SMAD3 and SMAD4 recognize and bind the identical palindromic promoter sequence during transcriptional regulation (14), this suggests that the effects we see on COL1A2 expression may simply be due to SMAD3 homodimers inhibiting the formation of transcriptionally active SMAD3/SMAD4 heterodimers.

In conclusion, AAV-based hSMAD3 gene therapy exceeded our expectations in providing the therapeutic “good face” of TGFβ1 over that of the bad (no fibrosis). hSMAD3-therapeutic gene delivery was successful in reducing the pathology of HCD, a prototype Western diet in LDLR KO mice, both in reducing atherogenesis and enhancing Th2 response. Yet, hSMAD3 delivery did this without inducing the serious TGFβ1-associated adverse side effect of fibrosis as measured by CTGF and collagen expression. As of now, our main hypothesis is that the effects of AAV/hSMAD3 gene delivery are driven by an increase in nuclear SMAD3 homodimers and their resulting changes in transcriptional regulation. Overall effects of hSMAD3 gene delivery were without documented or noticed side effects. However, atherosclerosis was not fully inhibited. Perhaps increasing the extent of hSMAD3 gene delivery, or improving it’s level of expression can result in further down-regulation of the disease state. Thus, further studies into the use of AAV-based hSMAD3 gene delivery are warranted.

## Conclusions

This animal study focused on the gene therapy manipulation of the very powerful TGFβ1 signal transduction pathway for inhibiting atherosclerosis, by the delivery of the human SMAD3 gene. SMAD3 is one of the transcription factors through which TGFβ1 acts. We found that AAV2/8-hSMAD3 delivery did give efficacy in inhibiting HCD-induced atherosclerosis in LDLR KO mice. Moreover, significantly increased fibrosis was not observed as is usually the case when direct, primary TGFβ1-signalling is stimulated. Previously, we have shown that, analogous to this study, AAV/STAT3 gene delivery, with STAT3 being down-stream of interleukin 10 (IL10), is similarly able to substitute for IL10, again, for inhibiting atherosclerosis. Thus, through this strategy of using downstream signal transduction genes in place of their powerful primary chemokines, we might be able to effect superior treatment results.
